# Circular RNAs modulate cancer drug resistance: advances and challenges

**DOI:** 10.20517/cdr.2024.195

**Published:** 2025-03-28

**Authors:** Jinghan Hua, Zhe Wang, Xiaoxun Cheng, Jiaojiao Dai, Ping Zhao

**Affiliations:** ^1^The First Affiliated Hospital, Zhejiang University School of Medicine, Hangzhou 310000, Zhejiang, China.; ^2^Institute of Clinical Pharmacology, Anhui Medical University, Hefei 230000, Anhui, China.; ^3^The Second Clinical School of Anhui Medical University, Hefei 230000, Anhui, China.

**Keywords:** Drug resistance, CircRNAs, cancer therapy, biomarkers, AI-based bioinformatics algorithms

## Abstract

Acquired drug resistance is a main factor contributing to cancer therapy failure and high cancer mortality, highlighting the necessity to develop novel intervention targets. Circular RNAs (circRNAs), an abundant class of RNA molecules with a closed loop structure, possess characteristics including high stability, which provide unique advantages in clinical application. Growing evidence indicates that aberrantly expressed circRNAs are associated with resistance against various cancer treatments, including targeted therapy, chemotherapy, radiotherapy, and immunotherapy. Therefore, targeting these aberrant circRNAs may offer a strategy to improve the efficiency of cancer therapy. Herein, we present a summary of the most recently studied circRNAs and their regulatory roles on cancer drug resistance. With the advances in artificial intelligence (AI)-based bioinformatics algorithms, circRNAs could emerge as promising biomarkers and intervention targets in cancer therapy.

## INTRODUCTION

Despite significant advances in cancer treatment over the decades, cancer remains one of the most clinically lethal diseases, mainly due to the development of acquired drug resistance^[1,2]^. Patients initially exhibit a high response rate to cancer treatment, with a noticeable suppression of cancer progression. However, acquired drug resistance develops when a substantial proportion of cancer cells become resistant to cancer treatment options, leading to cancer recurrence or progression^[1,3,4]^. A variety of factors contribute to the formation of drug-resistant cancer cells, such as tumor heterogeneity, tumor microenvironment and so on^[4-7]^. Therefore, it is urgent to develop novel intervention targets and effective strategies to reverse cancer drug resistance, which facilitates the clinical outcomes of cancer treatment.

Circular RNAs (circRNAs) are a special type of non-coding RNA molecules that form a circular conformation via non-canonical splicing or back-splicing events^[8]^. CircRNAs were initially considered by-products of transcription with low abundance^[8,9]^. With the rapid development of high-throughput sequencing and bioinformatics algorithms, thousands of circRNAs have been identified across a wide range of species, from viruses to mammals. Notably, aberrant expression of circRNAs in various diseases prompted further research into their potential regulatory functions. Accumulating evidence has demonstrated that circRNAs are associated with specific hallmarks of cancer, including sustaining proliferative signaling, evading growth suppressors, and activation of invasion and metastasis^[10]^. Moreover, a series of circRNAs with significantly altered expression following cancer treatment are involved in modulating cancer drug resistance^[11-13]^. Therefore, elucidating the functions and mechanisms of these circRNAs could provide a deeper understanding of cancer drug resistance.

In this review, we summarize the current research on circRNAs in various cancer treatment strategies, including resistance to targeted therapy, chemotherapy, radiotherapy, and immunotherapy. The development of emerging technologies, such as artificial intelligence (AI)-aided design, has greatly improved the efficiency of circRNA synthesis and delivery. Several challenges regarding the clinical translation and application of circRNAs are also discussed. We aim to shed new light on circRNAs-mediated cancer drug resistance and their translational potential as intervention targets for cancer therapy, developing clinical protocols for circRNAs-based therapies to overcome cancer drug resistance.

## BIOGENESIS AND REGULATORY ROLES OF CIRCRNAS

CircRNAs are a class of endogenous RNA molecules derived from precursor mRNA (pre-mRNA) via back-splicing, in which a downstream splice site is joined with an upstream splice site^[8]^. Reverse complementary sequences in the flanking regions of circularized exons, along with RNA-binding proteins, are the major factors contributing to circRNA biogenesis^[8,14]^. CircRNAs can be mainly divided into two groups based on their sequence origin. One group consists of cytosolic genome-derived circRNAs, including exonic circRNA (EcircRNA), exon-intron circRNA (EIciRNA), and intronic circRNA (CiRNA), while the other comprises the mitochondria-encoded circRNAs (MecciRNAs)^[6,15]^.

The function of circRNAs is associated with their subcellular location patterns. Generally, cytoplasmic circRNAs exert their regulatory roles by acting as microRNA sponges to remove the inhibitory effects on the targets, serving as RNA-binding protein decoys to modulate downstream biological processes and acting as templates for translation to generate functional polypeptides. In contrast, nucleus-localized circRNAs typically regulate the expression of target genes by affecting their transcriptional processes in the promoter region^[15]^.

## CIRCRNAS HOLD POTENTIAL AS BIOMARKERS FOR TUMOR DIAGNOSIS AND PROGNOSIS

**Figure 1:**
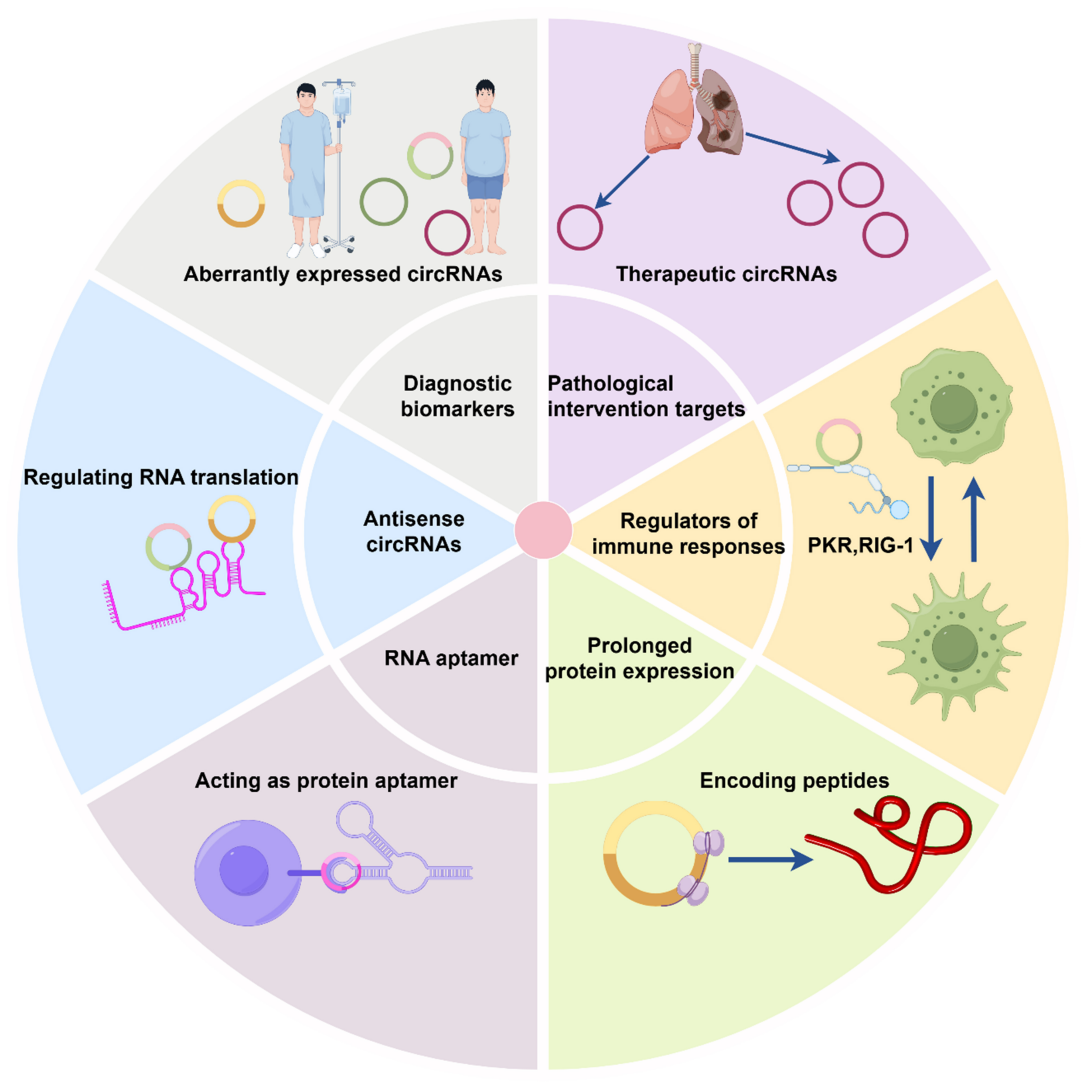
Clinical significance and application of circRNAs. This diagram illustrates the various roles and functions of circRNAs in biological systems. A large number of dysregulated circRNAs and their expression patterns are associated with the progression of multiple cancers, emerging as diagnostic biomarkers and pathological intervention targets. Additionally, circRNAs show the potential to serve as antisense RNAs to interfere with transcription processes, act as aptamers or translational templates to regulate protein expression, and either activate or inhibit immune responses. circRNAs: Circular RNAs.

Due to their covalently closed-loop structure and lack of exposed terminal ends, circRNAs are resistant to degradation by exonucleases and highly stable in blood and other body fluids^[14,16]^. As shown in Figure 1 and Table 1, numerous dysregulated circRNAs and their expression patterns are associated with the progression of multiple cancers, emerging as promising prognostic biomarkers and intervention targets^[17-38]^. A total of 59,056 circRNAs were identified from 122 pairs of clinical samples among seven solid tumors and their adjacent normal tissues, with dysregulated circRNAs exhibiting cancer-specific expression or shared common expression signatures across cancers^[17]^. Xu *et al.* conducted a plasma-derived liquid biopsy profiling to systematically screen upregulated circRNA candidates in pancreatic ductal adenocarcinoma (PDAC), and ultimately established a circRNAs-based biomarker panel in conjunction with cancer antigen 19-9 expression to enable noninvasive detection of early-stage PDAC^[18]^. Moreover, several consortium projects including The Cancer Genome Atlas (TCGA), ExoRBase, and CircRNADisease have provided vast circRNA sequencing data. DeepBase integrates extensive clinical data on cancer-associated circRNAs, and analyzes the correlation between circRNA expression and survival outcomes of cancer patients^[19]^.

**Table 1:** Clinicopathological characteristic of circRNAs in cancers

**CircRNA**	**Cancer type**	**Cases**	**Expression**	**TNM stage (I + II/III + IV)**
CircRTN4^[24]^	PDAC	87	Upregulation	73/14
CircCAPRIN1^[25]^	Colorectal cancer	259	Upregulation	95/164
CircPDHK1^[26]^	Clear cell renal cell carcinoma	148	Upregulation	97/51
CircARID1A^[27]^	Gastric cancer	21	Upregulation	3/18
CircNFIB^[28]^	Intrahepatic cholangiocarcinoma	182	Downregulation	63/119
CircSHKBP1^[29]^	Gastric cancer	76	Upregulation	34/118
CircCDR1as^[30]^	Gastric cancer	82	Downregulation	28/54
Circ_0018289^[31]^	Cervical cancer	192	Upregulation	119/73
Circ_0003159^[32]^	Gastric cancer	108	Downregulation	30/78
Circ_100876^[33]^	Non-small cell lung cancer	101	Upregulation	58/43
CircNRIP1^[34]^	Gastric cancer	80	Upregulation	40/40
CircUSP7^[35]^	Non-small cell lung cancer	126	Upregulation	61/65
Circ_0001006^[36]^	Triple-negative breast cancer	133	Upregulation	82/51
Circ_001988^[37]^	Colorectal cancer	31	Downregulation	15/16
Circ_0048764^[38]^	Breast cancer	41	Upregulation	27/14

circRNAs: Circular RNAs; TNM: tumor-node-metastasis; PDAC: pancreatic ductal adenocarcinoma.

Aberrant expression of regulatory circRNAs may directly or indirectly contribute to oncogenesis and progression. For instance, a downregulated circRERE acts as a competitive endogenous RNA against miR-6837-3p, alleviating the repressive effect on MAVS and suppressing colorectal cancer progression^[20]^. Li *et al.* reported that upregulated circLARP1B acts as a protein decoy for hnRNPD to weaken the stability of LKB1 mRNA, facilitating cellular lipid accumulation and hepatocellular carcinoma metastasis^[21]^. Additionally, activation of circular E-cadherin RNA encodes an oncogenic E-cadherin protein variant through multiple-round open reading frame (ORF) translation, enhancing EGFR-STAT3 signaling transduction and glioblastoma tumorigenicity^[22]^. Nuclear-localized circMYBL1 enhances the binding of CEBPB to CD44 promoter regions, promoting CD44 transcription and adenoid cystic carcinoma progression^[23]^.

## CIRCRNAS ARE WIDELY INVOLVED IN REGULATING CANCER DRUG RESISTANCE

Despite significant progress in cancer treatments, nearly 90% of cancer-related deaths are attributed to the emergence of cancer drug resistance or drug resistance^[39]^. Therefore, identifying novel regulatory targets that mediate cancer drug resistance is necessary to develop effective intervention strategies. CircRNAs are associated with multiple mechanisms of acquired cancer drug resistance, such as epithelial to mesenchymal transition, DNA damage repair, immune evasion, tumor-promoting inflammation, and genome instability^[40-43]^. As depicted in Table 2, circRNAs regulate various pathways involved in drug resistance, demonstrating strong potential in reversing drug resistance. Here, we illustrate the commonly proposed mechanisms by which circRNAs contribute to cancer drug resistance, with selected examples.

**Table 2:** CircRNAs regulate various pathways involved in drug resistance

**CircRNAs**	**Drug resistance**	**Mechanisms or pathway**
CircDCAF8	Regorafenib	Exosome transmission^[44]^
CircVAPA	BMS536924	MiR-377-3p & miR-494-3p/IGF1R^[45]^
CircMED27	Lenvatinib	MiR-655-3p/USP28^[46]^
CircESRP1	Cisplatin	MiR-93-5p/Smad7/p21-mediated epithelial-mesenchymal transition^[47]^
CircPDIA3	Oxaliplatin	CircPDIA3/miR-449a/XBP1 feedback loop^[48]^
CircATG4B	Oxaliplatin	Encoding circATG4B-222aa that prevents TMED10 from binding to ATG4B^[49]^
CircMETRN	Radiotherapy	MiR-4709-3p/GRB14/PDGFR^[50]^
CircCDYL2	Radiotherapy	RAD51-mediated homologous recombination repair capability^[51]^
CircNOP14	Radiotherapy	Ku70-dependent DNA damage repair^[52]^
CircKEAP1	Immunotherapy	IFN-γ^[53]^
CircNCOA3	Immunotherapy	MiR-203a-3p.1/CXCL1^[54]^
CircCCAR1	Immunotherapy	CircCCAR1/miR-127-5p/WTAP feedback loop^[55]^
CircPVT1	Chemotherapy	MiR-145-5p/ABCC1^[56]^
CircPVT1	Radiotherapy	MiR-1208/PI3K/AKT/mTOR^[57]^
CircPPAPDC1A	Osimertinib	MiR-30a-3p/IGF1R^[58]^
CircITGB6	Cisplatin	CircITGB6/IGF2BP2/FGF9 RNA-protein complex^[59]^

CircRNAs: Circular RNAs.

### CircRNAs and resistance to targeted therapy

Targeted therapy is an approach to inhibit tumor growth or metastasis by blocking cancer-specific genes and signaling pathways based on gene mutations/expression^[60-63]^. Compared with traditional chemotherapy, targeted therapy can specifically kill tumor cells with minimal impact on normal cells and reduce drug side effects^[64]^. It has been widely accepted that the high failure rate of clinical targeted therapy may be attributed to cancer drug resistance. However, the role of circRNAs in targeted therapy drug resistance remains to be fully elucidated. A series of studies have demonstrated that targeting circRNAs could sensitize cancer cells to cancer therapy. Regorafenib is a multi-targeted receptor tyrosine kinase inhibitor that shows high antitumor and anti-angiogenic activity in hepatocellular carcinoma^[65-67]^. Gong *et al.* reported that circDCAF8 potentiates regorafenib resistance through exosome transmission, providing a promising protocol of circDCAF8 inhibition in combination with regorafenib for treating regorafenib-resistant hepatocellular carcinoma^[44]^. Aberrant regulation of the IGF1R signaling pathway has been recognized as a well-established therapeutic target in small cell lung cancer^[68,69]^. Hua *et al.* revealed that circVAPA knockdown enhances the dual repressive role of miR-377-3p and miR-494-3p on IGF1R expression, ultimately inhibiting small cell lung cancer progression^[45]^. Zhang *et al.* demonstrated that upregulated circMED27 is correlated with poor prognosis of hepatocellular carcinoma and elevated lenvatinib resistance via the miR-655-3p/USP28 axis, concluding that circMED27 inhibition may represent a promising strategy to overcome lenvatinib resistance^[46]^.

### CircRNAs and chemotherapy resistance

Chemotherapy combats cancers by disrupting DNA/RNA/protein synthesis or function to induce apoptosis^[42,70-72]^. However, the susceptibility of chemotherapy resistance remains the leading cause of treatment failure^[73]^. Multiple complex etiologies associated with chemoresistance contribute to poor prognosis of cancer, such as the inhibition of pathways mediating cell death, promotion of damaged DNA repair, and reduction of cellular drug accumulation^[74]^. Recent studies indicate that circRNAs regulate chemotherapy response as potential therapeutic targets to overcome drug resistance and improve outcomes.

Cisplatin is a well-known chemotherapeutic drug used in the treatment of various cancers. It induces cancer cells apoptosis of cancer cells by interfering with DNA repair^[75-81]^. CircESRP1 expression is aberrantly downregulated in the cisplatin-resistant cells compared with the parental cisplatin-sensitive cells, enhancing cisplatin sensitivity of small cell lung cancer via the miR-93-5p/Smad7/p21(CDKN1A) axis^[47]^. Since circESRP1 overexpression has a potent inhibitory effect on epithelial-mesenchymal transition, Huang *et al.* suggested that circESRP1 overexpression may be a strategy to overcome chemotherapy resistance in small cell lung cancer^[47]^. Oxaliplatin is a platinum-based chemotherapeutic agent that induces DNA damage by forming intra- and interstrand crosslinks, widely used in colorectal cancer treatment^[82]^. However, the development of oxaliplatin resistance severely limits its clinical use and efficacy. CircPDIA3 is negatively associated with disease-free survival of colorectal cancer patients, inducing oxaliplatin resistance via circPDIA3/miR-449a/XBP1 feedback loop^[48]^. Pan *et al.* concluded that circATG4B-222aa, a novel protein encoded by circATG4B, could act as a decoy that prevents TMED10 from binding to ATG4B, ultimately contributing to increased autophagy and oxaliplatin resistance^[49]^.

### CircRNAs and radiotherapy resistance

Radiation therapy combats cancers by inducing DNA damage and triggering cell cycle arrest, senescence, and apoptosis via ionizing radiation^[83-86]^. CircRNAs have also been implicated in radiation therapy. For example, Wang *et al.* used RNA sequencing techniques to assess the expression profiles of aberrant circRNAs undergoing glioma radiotherapy, demonstrating that low-dose radiation-induced exosome circMETRN could promote DNA damage repair process and radiotherapy resistance in glioblastoma cells through the miR-4709-3p/GRB14/PDGFRα axis^[50]^. Qu *et al.* revealed that circCDYL2 facilitates the initiation of RAD51 translation and homologous recombination repair capability through recruitment of EIF3D protein to the 5’-untranslated regions (UTR) of RAD51 mRNA, ultimately leading to radiotherapy resistance in nasopharyngeal carcinoma^[51]^. Similarly, circNOP14 enhances the radiosensitivity of hepatocellular carcinoma cells via Ku70 interaction to suppress Ku70-dependent DNA repair, providing a potential therapeutic target for hepatocellular carcinoma radiotherapy^[52]^. These studies provide mechanistic insights into the roles of circRNAs and identify valuable markers for overcoming cancer radiotherapy resistance.

### CircRNAs and immunotherapy resistance

Tumor immunotherapy is an approach designed to activate the immune cells and enhance the antitumor immune response with minimal side effects, specifically targeting small residual tumor lesions and inhibiting tumor growth^[87-89]^. It is well known that RIG-I signaling is involved in the transcriptional activation and expression of multiple pro-inflammatory genes^[90-93]^. Zhang *et al.* revealed that N6-methyladenosine (m6A)-modified circKEAP1 could interact with RIG-I to activate antitumor immunity through the IFN-γ pathway, providing new insights into the immune response in osteosarcoma^[53]^. Significant clinical progress has been made in various therapies targeting immune checkpoint inhibitors including programmed death receptor 1 (PD1) and PD1 ligand 1 (PD-L1)^[94-96]^. For example, circNCOA3 is elevated in anti-PD-1-resistant colorectal cancer, functioning as a competing endogenous RNA to modulate CXCL1 expression and facilitate immune evasion^[54]^. Exosome-derived circCCAR1 accelerates the exhaustion of antitumor CD8^+^ T cells and promotes resistance to anti-PD1 therapy via the circCCAR1/miR-127-5p/WTAP feedback loop^[55]^. Zou *et al.* provided a new strategy to overcome deruxtecan resistance by inhibiting the interaction between the VDAC3-derived circRNA and HSPB1 protein^[97]^. CircRNAs exhibit low immunogenicity, mainly due to their covalently closed structure lacking a free 5’ cap and 3’ poly-A tail, a common pathogen-associated molecular pattern identified by innate immunosensors^[98-100]^. In addition, their endogenous source and stable exonuclease resistance minimize interactions with cytoplasmic RNA sensors, thereby reducing the risk of interferon activation. Notably, circRNA avoids the dsRNA-induced immune response observed in linear RNA. Yang *et al.* comprehensively summarized the progress and application of RNA vaccines in antitumor therapy, while outlining future directions for expanding this promising platform to a variety of therapeutic applications^[98]^. Li *et al.* highlighted the potential of exosomal circRNAs as diagnostic and prognostic predictive biomarkers, as well as a new strategy for clinical therapy^[99]^. Taken together, these findings demonstrate that circRNAs play a significant role in regulating immune therapeutic responses.

## CHALLENGES AND APPROACHES OF CIRCRNAS IN CLINICAL APPLICATION

### CircRNAs exhibit unique advantages in druggable transformation

A large body of clinical or experimental evidence suggests that circRNAs may serve as promising therapeutic targets to reverse cancer drug resistance^[19,100,101]^, providing new insights into expanding druggable targets from proteins to circRNAs. Based on data from the Gene Expression Omnibus (GEO) and Connectivity Graph (CMap) databases, Cao *et al.* identified 55 circRNAs for potential drug targeting and 2,802 circRNAs associated with drug resistance^[101]^. The ncRNADrug database curates validated drug resistance-associated circRNAs, drug-targeted circRNAs, and resistant cancer drug combinations, enabling the prediction of circRNA-drug resistance interactions^[101]^. The covalently closed-loop structure and the lack of exposed terminal ends enable circRNAs to evade cellular recognition as exogenous entities, thereby reducing their immunogenicity^[102,103]^. Drugs that target circRNAs, such as antisense oligonucleotides (ASOs) and small interfering RNAs (siRNAs), are RNA-based therapeutics that are not constrained by protein structures. These nucleic acid drugs exhibit advantages including broad druggable targets and high specificity. Multiple ASOs bound to circRNAs through complementary base pairing can effectively hinder their functions or expression. For instance, ASO-circSKA3 inhibits colorectal cancer progression by disrupting the SLUG-circSKA3 interaction, promoting SLUG ubiquitination and degradation^[104]^. Drugs can also disrupt circRNA-protein/RNA interactions or modulate circRNA stability. For example, circRNA-SORE is significantly upregulated in sorafenib-resistant hepatocellular carcinoma cells^[105]^. Mechanistically, circRNA-SORE binds to YBX1 and prevents PRP19-mediated ubiquitination and YBX1 degradation, thereby inducing sorafenib resistance. Silencing circRNA-SORE with specific siRNAs is shown to be effective in overcoming chemoresistance to sorafenib^[105]^. Overall, circRNAs exhibit characteristics such as high stability, abundance, and low immunogenicity, which make them particularly advantageous for cancer vaccines.

### Challenges of circRNAs in clinical application

As circRNAs gain more attention, regulatory circRNAs may emerge as novel nucleic acid drugs to overcome cancer drug resistance [Figure 1]. CircRNAs can function as RNA or protein aptamers, bind specifically to certain RNA molecules/proteins, and modulate the process of translating RNA into proteins. These circRNAs can be used as therapeutic tools to treat diseases. However, several challenges remain in the clinical translation of circRNAs, including their synthesis, purification, and delivery^[102,103]^.

The conventional synthesis of circRNAs *in vitro* mainly uses linearized plasmid DNA as a template for *in vitro* transcription to produce precursor RNA, which is then cyclized through chemical or enzymatic ligation^[106,107]^. Various factors, such as gene sequence variations and length constraints, adversely affect both the circularization efficiency and production scalability of these constructs, ultimately leading to suboptimal yields^[106,108]^. Moreover, it is difficult to separate circRNAs from linear RNA precursors and nicked RNAs of similar molecular weight, highlighting the urgent need to improve the purification efficiency and quality control of circRNA products^[109]^. Lipid nanoparticles (LNPs) are lipid-based nanoparticles that have been widely used as delivery vehicles for functional RNAs including mRNAs, siRNAs, and circRNAs^[110-118]^. However, there is a pressing need to substantially improve the modification and delivery tropism of LNPs to extend their application to extrahepatic tissues. Further development of novel nanoparticle systems is essential to improve the delivery efficiency and application range of circRNAs.

### Emerging technologies facilitate the clinical translation of circRNAs

Researchers have made notable progress on the above issues in the past decade. Large-scale single-cell transcriptomic studies have unveiled that a substantial proportion of circRNAs exhibit cell-specific expression patterns. Several databases serve as a critical resource for investigating the dynamic changes of circRNAs during embryonic development, tissue differentiation, and cancer biogenesis, while providing a unique and functional platform for the circRNA research community. The superior applicability of circRNAs as cell-type biomarkers in exploring tumor-infiltrating immune cell heterogeneity further underscores their essential biological roles in specific cellular contexts^[119]^. With the development of emerging technologies including AI-aided design, the clinical translation and application prospects of RNA-based therapeutics and drugs have significantly improved. Recently, a novel algorithm tool called circDesign was developed to optimize open reading frame (ORF) sequences, facilitating the circularization and translation of synthetic circRNAs, as well as enhancing the immune responses *in vivo*^[120]^. Lee *et al.* developed a circRNA engineering strategy involving end-to-end self-targeting and splicing reaction using *Tetrahymena* group I intron ribozyme, which effectively generates circRNAs *in vitro*^[106]^. To purify the circRNAs, candidate RNA bands matching the expected molecular weights were extracted by gel electrophoresis and digested with RNase R^[103,106]^. In addition, Wesselhoeft *et al.* combined SEC-HPLC with RNase R digestion to prepare circRNAs with a purity of 90%, significantly outperforming RNase R digestion alone^[121]^. This strategy further amplifies the differences between circRNAs, linear precursor RNAs, and nicked RNAs, improving the separation efficiency of circRNAs.

Several studies have reported advances in circRNA delivery systems. For instance, Qu *et al.* used the LNP system to prepare circRNA vaccines for the treatment of SARS-CoV-2 and its emerging variants^[122]^. The nano-delivery carrier LNPs are low-cost, highly stable, and can be produced on a large scale. However, some disadvantages of LNPs, including their tendency to accumulate in the liver and their limited extrahepatic application, should not be overlooked^[123-125]^. Xu *et al.* used tumor-tailored ionizable H1L1A1B3 LNPs to facilitate the delivery of IL-12 circRNAs, inducing a robust immune response in a Lewis lung carcinoma model and marked tumor regression, thereby broadening the prospects for circRNAs drug delivery in cancer therapy^[126]^. Mitochondria-targeting nanoparticle (Mito-NP) is a multifunctional encapsulated LNP that precisely delivers circSCAR to mitochondria in liver fibroblasts for the treatment of nonalcoholic steatohepatitis^[127]^. Exosomes, endogenous extracellular vehicles secreted by most cells, are emerging as promising drug delivery vehicles^[128-132]^. Yu *et al.* constructed rabies virus glycoprotein-circDYM-extracellular vesicles for targeted delivery of circRNAs across the blood-brain barrier, which inhibited microglial activation, reduced peripheral immune cell infiltration, and attenuated astrocyte dysfunction induced by chronic unpredictable stress^[133]^. Yang *et al.* demonstrated that engineered rabies virus glycoprotein-circSCMH1-extracellular vesicles promote functional recovery in rodent and primate ischemic stroke models, highlighting a promising clinical treatment strategy for stroke^[134]^.

## CONCLUSIONS AND PERSPECTIVES

Cancer drug resistance significantly limits the effectiveness of clinical cancer treatment, which has been a major focus of research. Considering that aberrant circRNAs contribute to drug resistance in multiple cancers, a systematic understanding of circRNAs in cancer drug resistance can help identify new therapeutic targets. We summarize several circRNAs that are aberrantly expressed during cancer treatment and discuss current knowledge of how circRNAs modulate cancer drug resistance through various underlying mechanisms. For example, circPVT1 promotes chemotherapy resistance in lung adenocarcinoma via miR-145-5p/ABCC1 axis, while it also decreases the radiosensitivity in non-small cell lung cancer cells by modulating the miR-1208/PI3K/AKT/mTOR signaling axis^[56,57]^. Identifying and constructing circRNAs-mediated regulatory networks could offer a potential approach to overcome cancer drug resistance.

mRNA-based drugs and therapeutics have been successfully applied in clinical practice^[122,126,134]^. However, their widespread use of mRNA drugs and therapeutics is still limited by factors such as low stability, short expression duration, and potential immunogenicity^[122,126,134]^. It is noteworthy that circRNAs possess several advantages, including high stability and being immunosilent. As shown in Figure 1, several circRNAs with aberrant expression can serve as biomarkers for various diseases. CircRNAs can also modulate the immune response, affecting how the body fights against infections and diseases. Some circRNAs have the potential to encode small peptides, which hold diverse biological functions. Moreover, antisense circRNAs may regulate gene expression by interacting with complementary RNA sequences or specific proteins, while their binding capacity could also potentially extend protein expression duration by delaying molecular degradation. Zhu *et al.* have revealed that gut microbiota can regulate tumor metastasis through circRNA/miRNA networks^[135]^. This circRNA/miRNA-dependent regulatory mechanism not only deepens our understanding of cancer progression, but also provides a theoretical foundation for future clinical interventions targeting gut microbiota. Notably, the back-spliced junction site of circRNAs plays vital roles in the identification and application of circRNAs. The putative back-spliced junction fragments of circRNAs can be amplified with divergent primers and confirmed by Sanger sequencing^[45]^. To reduce the impact on the corresponding linear transcripts, RNA interferences against circRNAs are usually designed to target the back-spliced junction site of circRNAs.

Multiple circRNAs are associated with the prognosis of cancer therapy, emerging as promising biomarkers or intervention targets for cancer drug resistance. For instance, an upregulation of nearly 138 circRNAs and a downregulation of 86 circRNAs have been observed in chemotherapy-resistant small cell lung cancer cells, indicating that circRNAs play critical roles in the modulation of cancer resistance^[47]^. Tang *et al.* discovered that circPPAPDC1A decreases the susceptibility of non-small cell lung cancer cells to osimertinib via the miR-30a-3p/IGF1R axis, suggesting that circPPAPDC1A may be an appropriate candidate for the treatment of osimertinib-resistant non-small cell lung cancer cases^[58]^. In another study by Li *et al.*, the circITGB6/IGF2BP2/FGF9 RNA-protein complex enhances cisplatin resistance in ovarian cancer cells by facilitating the polarization of tissue-associated macrophages toward M2 macrophages, providing a clear perspective for the treatment of cisplatin-resistant ovarian cancer patients^[59]^.

Although the role of circRNAs in cancer drug resistance in physiological and pathological conditions has been well established, the clinical translation of circRNAs is still in its infancy. Synthesis, purification, delivery, and quality control remain significant challenges for circRNAs-based drugs. While advances have been made in circRNAs-based therapeutics^[136]^, several challenges, including synthesis efficiency, delivery precision, pharmacokinetics, and biodistribution of circRNAs, remain to be addressed. Advances in emerging technologies, such as AI-based bioinformatic algorithms and integrated databases associated with drug resistance, have shown a significant role in improving the rational design, synthesis, and translation efficiency of circRNAs, as well as their application in overcoming tumor drug resistance. AI-based bioinformatics algorithms can assist researchers in identifying novel internal ribosome entry site (IRES) elements and optimize 5’ UTRs, ultimately enhancing circRNA translation efficiency^[137]^. Several databases have integrated both experimentally validated and computationally predicted ncRNAs associated with drug resistance, aiding the screening and identification of circRNAs linked to cancer drug resistance^[138,139]^. Expanding the depth of databases on circRNAs, as well as further innovation in AI-based bioinformatics algorithms, are essential to broaden the applicability of circRNAs.

In summary, circRNAs are promising regulators that mediate cancer drug resistance, while further multidisciplinary research is needed to maximize their potential in clinical therapeutic applications. Although circRNAs hold potential in modulating cancer treatment resistance mechanisms, their clinical application necessitates further multidisciplinary research to optimize target selection, delivery systems, and therapeutic validation across diverse tumor microenvironments.

## DECLARATIONS

### Copyright

© The Author(s) 2025.
